# 156 ’Cycle-IN’ Student facilitated cycle skills training for children with intellectual and developmental disabilities: An applied case study

**DOI:** 10.1093/eurpub/ckae114.029

**Published:** 2024-09-26

**Authors:** Erin Byrd, Gary McDermott, Laurence Taggart, Clare McMonagle

**Affiliations:** Ulster University, Derry, United Kingdom; Ulster University, Derry, United Kingdom; Ulster University, Derry, United Kingdom; Ulster University, Derry, United Kingdom

## Abstract

**Purpose:**

In an effort to address the discrepancies in opportunities for children and young people with intellectual and developmental disabilities (IDD) to develop fundamental skills and confidence riding a bicycle, a 12-week collaborative interdisciplinary student-led cycling programme ‘Cycle-IN’ was developed by Ulster University Faculty of Life and Health Sciences and Foyle Down Syndrome Trust.

**Methods:**

Framed within interpretive phenomenology, and drawing upon interviews conducted with the young cyclists, student facilitators, organisation staff and parents, this case study explores the lived experiences of the Cycle-IN programme and evaluates whether the programme is in fact assisting children with IDD (n = 10) to become more confident cyclists, and supporting the professional development of sport and health science student’s (n = 11) competence and confidence to work with children with additional challenges.

**Results:**

Results of this programme demonstrate an overall acceptability of the programme from multiple perspectives. Attendance at sessions was high for both children (M = 83.3% ± 19.7) and student volunteers (84.2% ± 10.0). Mean percentage improvement in cycle skills was 42.6% (±29.4) with mean percentage of basic cycle skills attained 61.6% (±34.3). Qualitative evaluation of the programme identified 1) the challenges and opportunities of the programme, 2) inputs and outputs necessary for sustainable implementation of the programme, and 3) overall recommendations for programme development and scalability into additional contexts, from the perspective of the children, students, organisation staff and parents or caregivers.

**Conclusion:**

This case study presents a novel approach to supporting physical activity and fundamental motor skill development in children with IDD, as well as providing undergraduate students with the opportunity to engage with populations who face additional challenges and barriers to movement. Practice implications are presented in relation to the fidelity of the programme, as well as the sustainability and scalability of such interventions across contexts.

**Support/Funding Source:**

This project was delivered through the Cycle-IN programme at Ulster University and supported by Foyle Down Syndrome Trust.

